# Strawberry Maturity Recognition Algorithm Combining Dark Channel Enhancement and YOLOv5

**DOI:** 10.3390/s22020419

**Published:** 2022-01-06

**Authors:** Youchen Fan, Shuya Zhang, Kai Feng, Kechang Qian, Yitong Wang, Shangzhi Qin

**Affiliations:** 1 School of Space Information, Space Engineering University, Beijing 101416, China; qiankechang@126.com; 2 School of Space Command, Space Engineering University, Beijing 101416, China; z19891687879@sina.com (S.Z.); fzsyk210112@sina.com (K.F.); wangyitong956@163.com (Y.W.); qinshangzhi@outlook.com (S.Q.)

**Keywords:** strawberry ripeness, dark channel de-fogging, all-day picking, bad fruit

## Abstract

Aiming at the problems of low accuracy of strawberry fruit picking and large rate of mispicking or missed picking, YOLOv5 combined with dark channel enhancement is proposed. In “Fengxiang” strawberry, the criterion of “bad fruit” is added to the conventional three criteria of ripeness, near-ripeness, and immaturity, because some of the bad fruits are close to the color of ripe fruits, but the fruits are small and dry. The training accuracy of the four kinds of strawberries with different ripeness is above 85%, and the testing accuracy is above 90%. Then, to meet the demand of all-day picking and address the problem of low illumination of images collected at night, an enhancement algorithm is proposed to enhance the images, which are recognized. We compare the actual detection results of the five enhancement algorithms, i.e., histogram equalization, Laplace transform, gamma transform, logarithmic variation, and dark channel enhancement processing under the different numbers of fruits, periods, and video tests. The results show that combined with dark channel enhancement, YOLOv5 has the highest recognition rate. Finally, the experimental results demonstrate that YOLOv5 is better than SSD, DSSD, and EfficientDet in terms of recognition accuracy, and the correct rate can reach more than 90%. Meanwhile, the method has good robustness in complex environments such as partial occlusion and multiple fruits.

## 1. Introduction

Strawberry [1] is a general term for strawberry plants of Rosaceae. Strawberry fruit is conical with bright red color, while flesh color is light red with medium hardness. Having high and stable sugar in fruit and tasting sweet, sometimes a little sour, strawberry has a broad audience in China and has a high economic efficiency among agricultural products. However, there is not peel protect its soft flesh which is so easy for it to change color and even deteriorate. So, strawberries must be picked and sold in time after maturity. In the large-scale production of strawberries, the accurate and efficient identification of strawberry maturity is particularly important. Under the trend of agricultural modernization in China, compared with the traditional manual sensory recognition and picking, equipment with the technology of accurate and efficient automatic identification and picking becomes key. 

Some studies on the rapid identification of strawberry maturity are as follows. Using image technology combined with hyperspectral imaging [2], Jiang Hao extracted the ROI (the “region of interest” of strawberry hyperspectral images defined for accurate extraction of spectral data) average spectrum of strawberry samples and combined it with Fisher’s linear discriminant method to establish a model for identifying strawberry maturity, even finding the hyperspectral parameters with higher recognition accuracy. Using the HIS color space model [3], Zhao Ling extracted the color histogram of strawberry images’ H component, combined it with BP neural network, and established the strawberry maturity detection model based on color. Its accuracy reached more than 90%. But its shortcomings also lie in the fact that the detection model established by using HIS color space requires high illumination, and the effective recognition of strawberry ripeness cannot be completed under the condition of low illumination. The algorithm in this paper addresses the shortcomings of the existing technology by considering and improving the following three aspects: fruit category perfection, spectral band limitation, and environmental conditions.

In the previous literature, strawberries are mainly divided into three categories according to the coloration of the fruit surface: (1) immature one, of which more than 90% is cyan and the remainder is milky white; (2) fruit that is close to maturity, of which more than 80% is milky white and the remainder is red; (3) mature ones, coloring more than 80% bright red. However, the reality is that some bad fruits are similar in color to mature strawberries. Therefore, based on original classification, a class called bad fruit is added according to the size and fullness of the fruit, in which fruit is small and shriveled with dark red or black color. The targeted addition of the category of “bad fruit” improves the completeness and usability of the model and reduces the misjudgment of ripe fruit in the process of recognition. Secondly, compared with the existing strawberry recognition techniques, this paper chooses the color space model instead of the spectral feature model as the basis so that the model is not limited by the spectral feature band and is more universal. Meanwhile, taking the influence of all-day light on the images’ color model into account, based on the YOLOv5 algorithm, the enhancement algorithm of dark channel defogging is used to improve the problem of the influence on the color of the collected images at night with low illumination to enhance the accuracy of strawberry recognition at night.

## 2. Materials and Methods

### 2.1. Image Acquisition

This study was conducted on “Fengxiang” strawberry grown in Huairou District, Beijing. The strawberries were photographed continuously and randomly during a week from 13:00 to 14:00 when natural light was abundant and from 19:00 to 20:00 when natural light was weak, under the conditions of down light, back light, single fruit, multiple fruit and shade.

### 2.2. YOLOv5 Model

**Figure 1:**
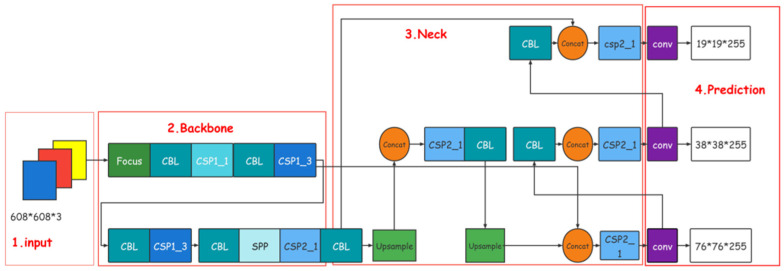
Network structure of YOLOv5.

The YOLO series is a single-stage target detection algorithm [4], and this study uses the YOLOv5 algorithm [5,6] proposed after several improvements of the series. YOLOv5, based on YOLOv4 [7,8], inherits the advantages while adding functions to improve the detection speed while ensuring accuracy. It is usually divided into four general modules, specifically: Input, Backbone, Neck, and Prediction. The network structure of YOLOv5 is shown in Figure 1.

YOLOv5 is subdivided into four versions, according to the network model from small to large, YOLOv5s, YOLOv5m, YOLOv5l, and YOLOv5x, according to the number of residual structures it contains in increasing order; the network’s feature extraction and fusion capabilities have been strengthened, and its detection accuracy is getting higher, but the speed is getting lower. The differences between the four network structures of YOLOv5 are shown in Table 1.

## 3. Image Preprocessing

### 3.1. Original Dataset

**Figure 2:**
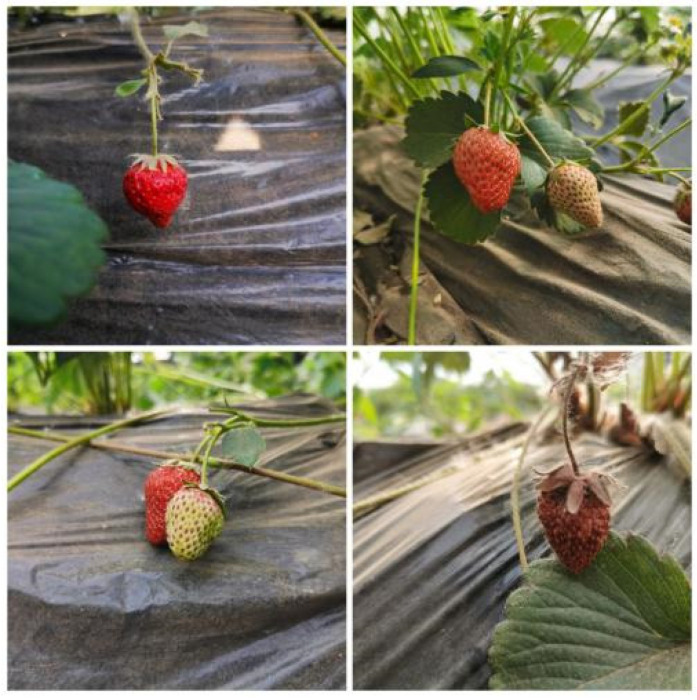
Original dataset of strawberry.

In deep learning methods, richer training data means the ability to train better models with deeper networks. In this study, the images of strawberries were divided into two categories: single fruit and multi-fruit, including 1000 and 1400 images, respectively. The single fruit images also include 260 mature strawberry images, 200 near mature strawberry images, 260 immature strawberry images, and 280 bad fruit images. Some of the original datasets are shown in Figure 2.

### 3.2. Image Data Amplification

This study utilizes the image level pixel enhancement (Mosaic data enhancement) built into YOLOv5 to enrich the dataset using four images in the form of random scaling, random cropping, and random rows, which greatly improves the training speed of the network and reduces the memory requirements of the model.

### 3.3. Image Marking

Strawberry image labeling uses the image labeling tool LabelImg to capture the strawberry target in the image as a training dataset, highlighting the image so that the image can be read by the machine. LabelImg is a graphical image annotation tool written by Python, which is stored into XML files according to the PASCAL VOC format used by ImageNet.

In the marking process, the strawberry target was manually marked by creating a box and a coordinate axis. The category labels 1 to 4 represent the four strawberry classifications of immature, near-mature, mature, and bad fruit.

## 4. Image Training

### 4.1. Training Environment

The hardware and software configurations used in the experiment are shown in Table 2 below. In this training environment, the batch_size of running the YOLOv5 program is set to 32, and the ratio of the training set, validation set, and test set is set to 6:2:2.

### 4.2. Training Results

**Figure 3:**
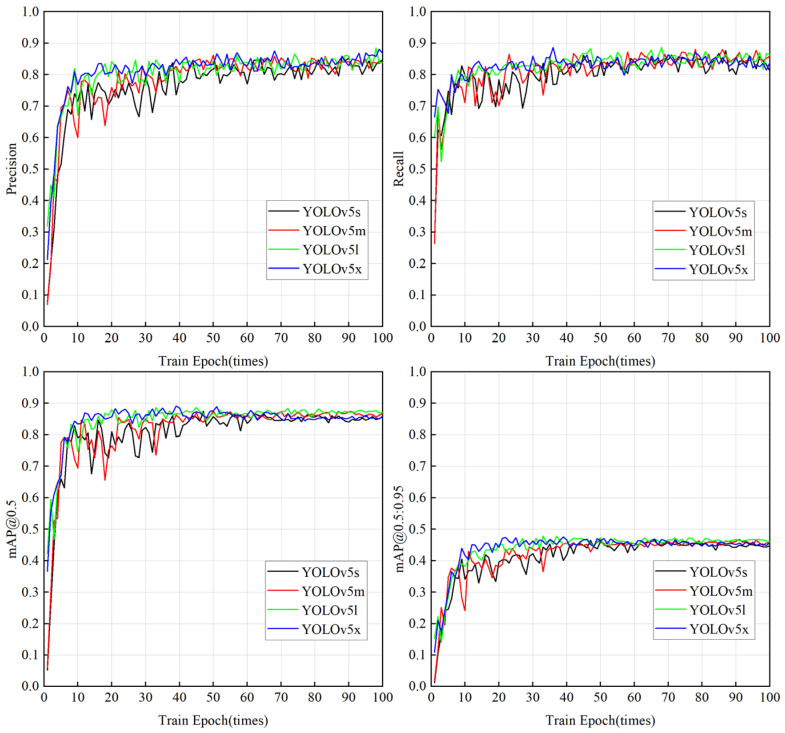
YOLOv5 training results.

The annotated training set and validation set images are trained 100 times in YOLOv5s, YOLOv5m, YOLOv5l, and YOLOv5x network models, respectively. The parameter results of the strawberry maturity recognition model are shown in Figure 3.

Comparing the training results under the four models of YOLOv5, we can more intuitively reflect the differences in weights trained by different network models. From the training results, it can be seen that with the increase in training times, the precision and recall rate of the four models are gradually stabilized at more than 80%, indicating that the precision and recall rate of the four models have reached a high level. When the IOU threshold is 0.5, the average accuracy of all strawberry categories (mAP, Mean Average Precision) is greatly improved and can be stabilized at more than 85%; when the threshold range of IOU is 0.5–0.95, the mAP value can be stabilized at about 45%. During the training process, the mAP value of YOLOv5x changes gently, which can keep the accuracy stable in the fastest time. The mAP value of YOLOv5s changes most violently, and the accuracy of the model is the most difficult to maintain stability when the number of trainings is small.

## 5. Comparison of Low-Illumination Enhancement Algorithms

To meet the requirements of all-day picking, low-illumination enhancement of strawberry images collected at night is required. In this study, histogram equalization [9,10,11,12], Laplace transform, gamma transform, logarithmic variation, and dark channel enhancement [13,14] will be applied to single-fruit and multi-fruit strawberry images under a nighttime environment, and four evaluation metrics, SSIM, peak signal-to-noise ratio, UCIQE, and mean variance, will be used to compare each of these five commonly used image enhancement algorithms.

### 5.1. Image Demonstration

**Figure 4:**
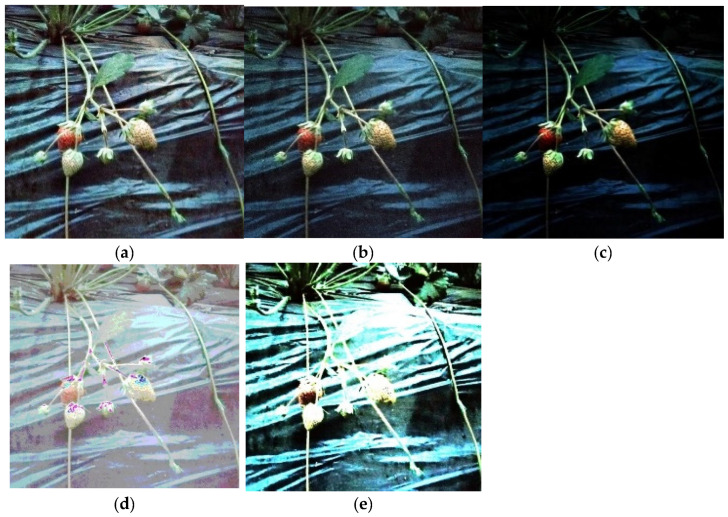
Enhanced image effect. (**a**) Histogram equalization; (**b**) Laplace transform; (**c**) Gamma transform; (**d**) Log transform; (**e**) Dark channel enhancement.

With the help of OpenCV module and Numpy module, the strawberry images processed by the five enhancement algorithms are shown in Figure 4.

### 5.2. Indicator Analysis

Four evaluation indexes, SSIM, peak signal-to-noise ratio, UCIQE, and mean variance, were used to calculate the index values of the five enhancement algorithms for nighttime low-light images after processing and judge the enhancement algorithm with the best effect. The index values are shown in Table 3.

(1) Structural similarity (SSIM) is a measure of the similarity of two images. By comparing the images before and after enhancement, it can be judged whether the enhancement changes the image greatly. The greater the structural similarity, the better the enhancement algorithm; conversely, the enhancement algorithm does not modify the original image information too much. The calculation formula is as follows.

(1) $$\mathrm{SSIM}\left(x,y\right)=\frac{\left(2\mu_{x}\mu_{y}+c_{1}\right)\left(2\sigma_{xy}+c_{2}\right)}{\left(\mu_{x}^{2}+\mu_{y}^{2}+c_{1}\right)\left(\sigma_{x}^{2}+\sigma_{y}^{2}+c_{2}\right)}$$

From the analysis of the data in the table, the enhancement effect of dark channel defogging on both single and multiple fruits was optimal and had a large gap with the remaining four, histogram equalization was the second most effective, and Log enhancement was the worst.

(2) Peak signal-to-noise ratio is a ratio of the maximum possible power of a representative signal and the destructive noise power that affects its representation accuracy. The peak signal-to-noise ratio is defined as

(2) $$PSNR=10\cdot \log_{10}\left(\frac{MAX_{I}^{2}}{MSE}\right)=20\cdot \log_{10}\left(\frac{MAX_{I}}{\sqrt{MSE}}\right).$$

The difference between the peak S/N ratio of the enhanced image and the original image is calculated here. The larger the difference between the peak S/N ratio, the better the enhancement. From the analysis of the data in the table, it is obtained that Laplace enhancement is optimal for both single and multi-fruit enhancement, with dark channel enhancement being the second most effective and Log transform enhancement being the worst.

(3) *UCIQE* is a reference-free underwater image quality evaluation index based on the excitation of human eye visual system. The calculation formula is as follows.

(3) $$UCIQE=c_{1}*\sigma_{c}+c_{2}*{\mathrm{con}}_{1}+c_{3}*\mu_{s}$$

The larger its value, the better the color balance, sharpness, and contrast of the image. From the analysis of the data in the table, the histogram equalization has the best enhancement effect for both single and multiple fruits, the dark channel enhancement effect is the second best and the difference between the two is not large, and the Laplace enhancement effect is the worst and the difference is obvious.

(4) Two *m* × *n* monochrome images *I* and *K*; if one is a noise approximation of the other, the mean squared error (*MSE*) is defined as


(4) $$MSE=\frac{1}{mn}\Sigma_{i=0}^{m-1}\Sigma_{j=0}^{n-1}{\left[I(i,j\right)-K(i,j)]}^{2}.$$

For the original image and the enhanced image, the larger the pixel mean squared error, the worse the enhancement effect, while conversely, the better the enhancement effect. From the analysis of the data in the table, Laplace enhancement is optimal for both single-fruit and multi-fruit enhancement, and the dark channel enhancement is the second most effective, and the gap between the two and the remaining three is larger, with Log transform enhancement being the worst.

### 5.3. Comparison of Enhancement Algorithms Conclusion

The dark channel enhancement and histogram equalization enhancement basically restore the visual effect under illumination conditions and effectively change the gray value of each region, which meets the basic requirements of human vision, but analyzing the evaluation indexes of both, the dark channel enhancement effect is better than the histogram equalization enhancement; the Laplace enhancement effect is slightly worse, which is reflected in its enhancement of local shadows, and the Laplace method. The effect of Laplacian enhancement is relatively poor, which is reflected in its enhancement of local shadows; the Laplacian method does not deal with shadows well but smooths them. The effect of Gamma enhancement was extremely insignificant and only enhanced partial green leaves. The Log transform enhancement focuses too much on the increase in gray value, which makes it impossible to distinguish the edges of the contour of the objects, and the overlapping phenomenon exists between the objects, which does not meet the requirements.

Considering the above factors, the optimal method “dark channel enhancement” was selected to enhance the strawberry images at night with low illumination.

## 6. Experiment Results and Analysis

### 6.1. Evaluation of Experimental Results of Four Network Structures

Using the models trained under the four network structures to check out the test set respectively, we take the averaged value in the pictures’ data of a single fruit test, multi-fruit test, test in sufficient light, and test at night. The results are shown in Table 4.

According to the data in Table 4, no matter what network structure is selected for training, the obtained model can effectively and accurately evaluate the maturity of strawberries. There are still differences in the models trained under different network structures. In terms of the time used for identification, the increasing order of models is YOLOv5s, YOLOv5m, YOLOv5l, and YOLOv5x. In terms of the accuracy of identification, the descending order of models is YOLOv5m, YOLOv5x, YOLOv5l, and YOLOv5s. The highest accuracy reaches 0.91, and the remaining ones also over 0.80. Among them, YOLOv5m is the optimal network structure for its fast time and best accuracy of recognition.

### 6.2. Performance Evaluation of Several Single-Stage Detection Methods

**Figure 5:**
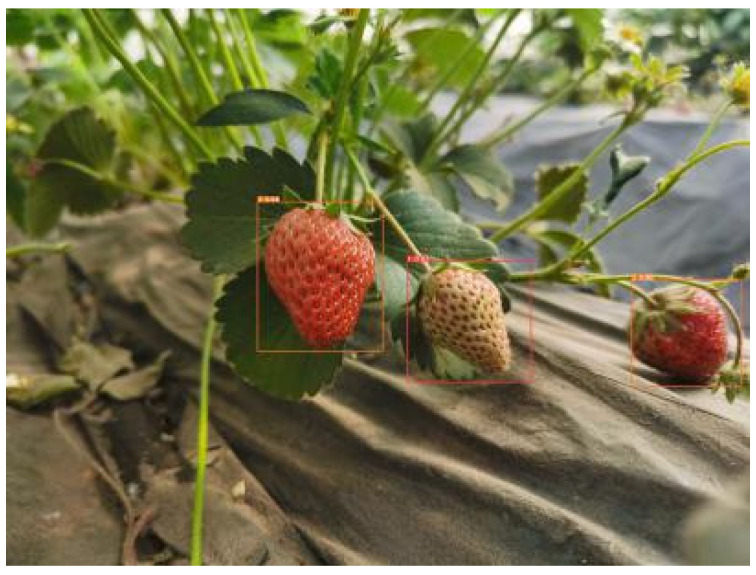
YOLOv5 strawberry maturity test renderings.

When YOLOv5m is used to train the dataset, the image test results of the four maturity levels of strawberries in various environments are averaged. According to the model test situation, the following results can be obtained, as shown in Table 4, and some of the test results are shown in Figure 5.

According to the data in Table 5, the model trained using YOLOv5m has at least 90% recognition accuracy for the four types of strawberries, and the difference between the four types is not obvious.

For the same dataset, three other classical single-stage detection methods SSD [15], DSSD [16], and EfficientDet [17] are selected for training, and the test results are shown in Table 6 below.

After comparing the model test results of the above three single-stage detection methods—SSD, DSSD, and EfficientDet—with the YOLOv5 test results, we found that the test results of YOLOv5 were much higher than the remaining three in terms of recognition accuracy. Therefore, for the category detection of strawberry maturity, choosing the YOLOv5 detection method can achieve better target recognition results.

### 6.3. Evaluation of the Effect of Dark Channel Enhancement Processing

The comparison of the model test results before and after the dark channel enhancement process for the low-illumination images of the test set is shown in Table 7 below.

From the data in Table 7, it can be obtained that comparing the test results before and after the dark channel enhancement process for nighttime low-light pictures, the recognition accuracy after enhancement is significantly higher than the accuracy before enhancement, and the accuracy can reach more than 0.80.

**Figure 6:**
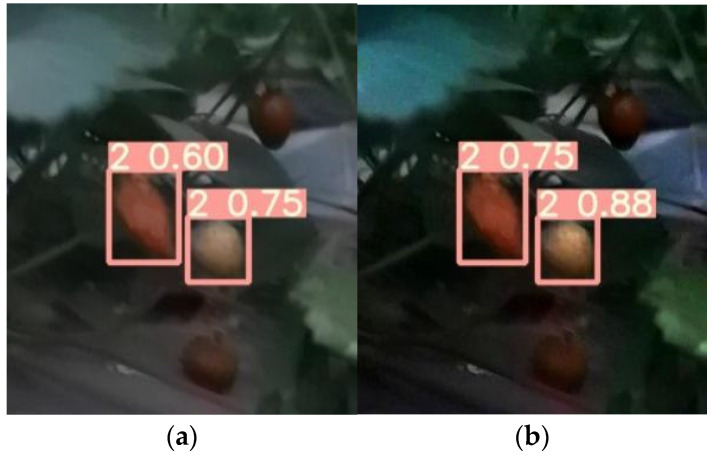
Comparison of the test results. (**a**) Test results for unenhanced images; (**b**) Test results of the enhanced image.

**Figure 7:**
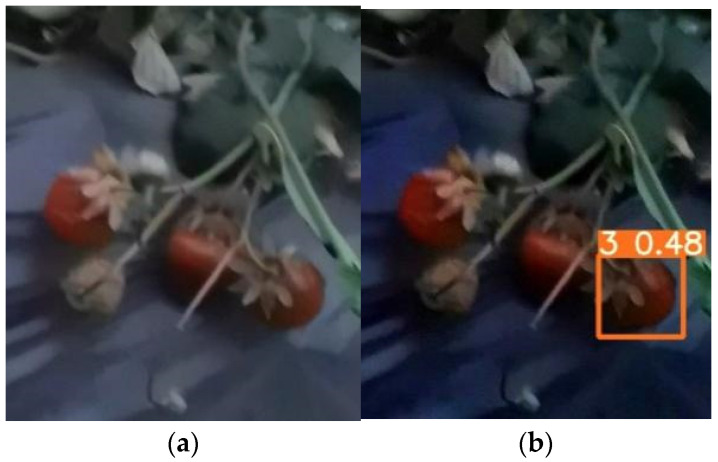
Comparison of test results before and after enhancement. (**a**) Test results for unenhanced images; (**b**) Test results of the enhanced image.

In the test set, we chose one original strawberry image at night that can be recognized and one that cannot be recognized before the dark channel enhancement processing. Then, we compared the changes of the test results before and after the dark channel enhancement processing. The comparison of the test results is shown in Figure 6 and Figure 7.

From the comparison of the test results before and after enhancement, it can be seen that for some multi-fruit images that can be incompletely recognized by the model at night are correctly predicted. It shows the dark channel enhancement process can further improve the execution of the detected strawberry fruit to a certain extent. For some unrecognizable images at night, the dark channel enhancement process enables individual fruits to be correctly predicted, which improves the accuracy and recall rate of model detection to a certain extent.

In summary, the selection of YOLOv5 combined with the recognition algorithm of dark channel can enhance the recognition rate and accuracy of strawberry ripeness and improve the problem of low illumination of images collected at night. Compared with the single-stage detection methods SSD, DSSD, and EfficientDet, YOLOv5 has the advantage of detecting high accuracy for the class detection problem of strawberry ripeness. Among the four network structure choices, YOLOv5m takes faster time and has the best recognition accuracy, and it is the optimal network structure choice with at least 0.90 recognition accuracy for all four ripeness of strawberries.

## 7. Conclusions

To improve the accuracy of strawberry identification and picking, this paper established four standards, i.e., mature, near-mature, immature, and bad fruit. Then, we proposed to add the dark channel recognition algorithm in YOLOv5. The experiments demonstrated that the proposed method can improve the accuracy of night recognition, which resulted in achieving strawberry recognition all day, high precision, high efficiency, and high robustness. Compared with the existing strawberry automatic rapid identification technology, the algorithm in this paper was more universal, has a wide range of applications, a larger time range for picking fruits, and relatively low requirements for environmental conditions such as light and shading. In the future, the dataset capacity can be further increased by expanding the number of samples or using more data amplification techniques, choosing different image enhancement methods for different periods of light conditions, and overlaying image enhancement methods to further improve the efficiency and accuracy of algorithm recognition rate. In combination with the requirements of field strawberry picking, in further research, we will consider transplanting this algorithm into the picking robot hardware, combining it with the robotic arm, to achieve the purpose of automatically identifying picking all day long.

## Figures and Tables

**Table 1:** YOLOv5’s four network structure differences.

Network Structure	Number of Residual Components (pcs)	Number of Convolution Kernels (pcs)
YOLOv5s	12	1001
YOLOv5m	24	1488
YOLOv5l	36	1984
YOLOv5x	48	2180

**Table 2:** Configuration of hardware and software.

Hardware or Software	Technical Parameter
operating system	Window 10 × 64 Home
GPU	NVIDIAGeForceRTX-3090
CPU	Intel(R)Xeon(R)Silver4116
memory	32 GB
deep learning library	TensorFlow
marking software	Labelimg
programming language	Python

**Table 3:** Comparison of enhancement effects.

	Adaptive Histograms	Laplace Transform	Gamma Transform	Log Transform	Dark Channel Enhancement
**SSIM**	0.65	0.63	0.28	0.23	0.85
**PSNR**	16	29	21	7	26
**UCIQE**	0.07	0.003	0.04	0.007	0.06
**MSE**	3960	82	1408	34,109	425

**Table 4:** Test results for different YOLOv5 models.

Network Structure	YOLOv5s	YOLOv5m	YOLOv5l	YOLOv5x
Time/s	0.1423	0.1439	0.1472	0.1527
Recognition accuracy	0.81	0.91	0.83	0.85

**Table 5:** Test results of strawberries of different maturity categories.

Classification of Strawberry Maturity	1 (Unripe)	2 (Almost Ripe)	3 (Ripe)	4 (Bad Fruit)
Recognition Accuracy	0.92	0.90	0.90	0.91

**Table 6:** Recognition accuracy results.

Classification of Strawberry Maturity	1 (Unripe)	2 (Almost Ripe)	3 (Ripe)	4 (Bad Fruit)
SSD	0.62	0.66	0.80	0.71
DSSD	0.72	0.73	0.83	0.76
EfficientDet	0.70	0.78	0.81	0.75

**Table 7:** Dark channel enhancement effect test in YOLOv5.

Classification of Strawberry Maturity	1 (Unripe)	2 (Almost Ripe)	3 (Ripe)	4 (Bad Fruit)
Accuracy before Enhancement	0.81	0.68	0.68	0.70
Accuracy after Enhancement	0.88	0.82	0.84	0.80
